# 固相支撑液液萃取结合超高效液相色谱-串联质谱快速检测牧草中10种麦角生物碱毒素

**DOI:** 10.3724/SP.J.1123.2025.02014

**Published:** 2026-04-08

**Authors:** Caixia REN, Xiaomin WANG, Wanli SHENG, Yizhi HAN, Chunyan ZHANG, Shuzhan ZHENG

**Affiliations:** 呼和浩特海关技术中心，内蒙古 呼和浩特 010020; Technical Center of Hohhot Customs，Hohhot 010020，China

**Keywords:** 麦角生物碱毒素, 牧草, 固相支撑液液萃取, 超高效液相色谱-串联质谱, ergot alkaloid toxins, forage grass, solid-phase supported liquid-liquid extraction, ultra performance liquid chromatography-tandem mass spectrometry （UPLC-MS/MS）

## Abstract

基于固相支撑液液萃取技术和超高效液相色谱-串联质谱法，建立了一种快速、灵敏检测牧草中10种麦角生物碱毒素的方法。牧草样品使用含1%甲酸的乙腈充分提取，取1.0 mL提取溶剂过固相支撑液液萃取柱净化，乙酸乙酯洗脱，浓缩后复溶为待测液。使用Acquity UPLC BEH C_18_色谱柱（100 mm×2.1 mm，1.7 μm）分离，以0.25 mmol/L乙酸铵溶液（含0.5%甲酸）和乙腈（含0.5%甲酸）为流动相进行梯度洗脱。采用电喷雾电离正离子（ESI^+^）扫描和多反应监测（MRM）方式检测，基质标准曲线外标法定量分析。结果显示，10种麦角生物碱毒素在各自线性范围内线性关系良好（*r* ^2^>0.995），在苜蓿、羊草、燕麦、玉米青贮等牧草中10种麦角生物碱毒素的检出限为0.1~2.3 μg/kg，定量限为0.4~7.3 μg/kg，方法的总体平均回收率为66.3%~116.7%，总体相对标准偏差小于9.9%，方法基质效应较低，净化效果明显，具有操作简单、结果准确、干扰少的特点，适用于牧草中麦角生物碱毒素的同步筛查和确证检测，可为监控牧草品质提供技术支持，同时拓展了固相支撑液液萃取技术在毒素检测领域的应用。

## Abstract (en)

Toxic alkaloids， especially ergot alkaloid toxins， present in forage grass and pose serious health hazards to humans and livestock. Hence， a method for simultaneously detecting these dangerous plant toxins is needed. This study established a rapid method for detecting ten ergot alkaloid toxins using solid-phase supported liquid-liquid extraction technology combined with ultra performance liquid chromatography-tandem mass spectrometry. Forage samples were extracted with 8.0 mL of acetonitrile containing 1% formic acid. The extraction mixture was vortexed and then centrifuged at 8 000 r/min for 5 min， after which 1.0 mL of the supernatant was mixed with 3.0 mL of water and vortexed for 0.5 min. The entire mixture was transferred to a solid-phase supported liquid-liquid extraction column and allowed to stand for 10 min. The column was eluted with 6.0 mL of ethyl acetate and the collected eluate was evaporated under nitrogen at temperatures below 40 ℃. The residue was dissolved in 1 mL of acetonitrile-water （1∶3， volume ratio， containing 0.5% formic acid）， and separated using an Acquity UPLC BEH C_18_ column （100 mm×2.1 mm， 1.7 μm）. A solution of 0.5% formic acid in water containing 0.25 mmol/L ammonium acetate was used as mobile phase A， while 0.5% formic acid in acetonitrile was used as mobile phase B. The following gradient elution program was used： 0-1.0 min， 90%B； 1.0-5.0 min， 90%B-75%B； 5.0-10 min， 75%B-50%B； 10-12 min， 50%B-10%B； 12-14 min， 10%B； 14-16 min， 10%B-90%B. Ten ergot alkaloid toxins （including three pairs of isomers： ergocornine， ergocristine， ergocristinine， ergocryptine， and ergocryptinine） were effectively separated following gradient elution. Positive electrospray-ionization mode and multiple reaction monitoring （MRM） scanning was used for detection， with an external standard curve used for quantification purposes. The ten ergot alkaloid toxins exhibited good linear relationships within their respective linear ranges （*r* ^2^>0.995）. Alfalfa， *Leymus chinensis*， oats， and silage corn exhibited LODs of 0.1–2.3 μg/kg for the ten ergot alkaloid toxins， with LOQs of 0.4–7.3 μg/kg. The developed method exhibited overall recovery rates of between 66.3% and 116.7%， with relative standard deviations of less than 9.9%. The matrix effect mainly manifested itself in the form of matrix suppression， with silage corn exhibiting a significantly stronger matrix effect than the other three types of forage. Silage corn exhibited a strong matrix effect in more than 50% of samples， while alfalfa， oats， and *Leymus chinensis* showed relatively low matrix effects， with more than 63% being weak. The developed method is simple to operate， provides accurate results， and exhibits minimal interference； hence， it is suitable for simultaneously screening and confirming ergot alkaloid toxins in forage， thereby providing technical support for monitoring forage quality， while also expanding the applicability of solid-phase supported liquid-liquid extraction technology in the toxin-detection field.

优质牧草是畜牧业发展的基石，而真菌毒素污染是威胁牧草品质的主要风险因素之一^［1，2］^，牧草中的真菌毒素污染种类复杂，除黄曲霉毒素类、呕吐毒素、玉米赤霉烯酮等常见毒素外，麦角生物碱毒素（如麦角柯宁碱、麦角克碱、麦角生碱、麦角胺等）因麦角菌感染植物而产生，可引发牲畜中枢神经系统损伤、流产、坏疽等严重病症，其污染问题备受关注^［3］^。Qie等^［4］^对内蒙古地区134份牧草样品进行了检测，20.19%的样本检出麦角生物碱毒素，其中检出率较高的项目为麦角克碱、麦角异克碱、麦角异卡里碱和麦角卡里碱。Di Mavungu等^［5］^检测了比利时122份饲草样本，其中牧草中70%检出麦角生物碱毒素，麦角胺最高含量350 μg/kg。Shi等^［6］^分析大麦和小麦中麦角生物碱毒素，麦角克碱为最主要检出物，大麦中检出率77%，含量最高达12 416.2 μg/kg。总体上看，麦角生物碱毒素污染比较普遍。尽管鲜有人类麦角毒素中毒的报道，但仍有牛、猪等动物中毒死亡的病例出现^［7，8］^，因此亟需建立高效检测方法以监控牧草安全。

文献报道的麦角生物碱毒素的检测方法主要有薄层色谱-荧光检测法（TLC-FLD）、毛细管电泳法（CE）和超高效液相色谱-串联质谱法（UPLC-MS/MS）等^［9，10］^，其中TLC和CE由于易受背景干扰，灵敏度和重复性不好，应用较少。UPLC-MS/MS具有分离速度快和灵敏度高的特点，因此目前真菌毒素残留检测中多采用适当的前处理技术结合UPLC-MS/MS的方法^［11，12］^。涉及牧草中麦角生物碱毒素检测的前处理技术报道较少，主要有液液萃取法（LLE）^［5］^、固相萃取法（SPE）^［13，14］^、QuEChERS法等^［4，6，15，16］^。Di Mavungu等^［5］^使用传统的液液萃取法处理提取液用以分析6种麦角生物碱毒素，方法有机溶剂使用量较大，不环保，且样品以谷物和谷物制品为主，牧草仅涉及青贮玉米一种；Qie等^［4］^采用QuEChERS法检测牧草中的生物碱，15种生物碱中仅有4种显示较弱基质效应，净化效果不够理想。Shi等^［6］^和汪薇等^［15］^均采用QuEChERS法分析麦角生物碱毒素残留，但都针对谷物，未涉及牧草样品，也缺少基质效应研究。综上所述，对于牧草中麦角生物碱毒素检测的前处理技术仍亟待拓展。

固相支撑液液萃取法（solid-phase supported liquid-liquid extraction， SLE）在20世纪80年代作为传统液液萃取方法的改进方法被提出^［17］^，该方法以改性硅藻土作为支撑材料，材料自身的多孔结构能够对水相有较强的吸附，可在表面形成高比表面积的薄液膜，利用薄液膜实现两相间快速高效的液液萃取。与传统液液萃取法相比，SLE萃取效率显著提高，且不产生乳化现象；与固相萃取法相比，避免了萃取柱活化和杂质淋洗等步骤，仅需要上样、洗脱两步，操作极大简化。SLE在生物样本中的药物检测和环境污染物分析以及牛奶中农药残留和饲料中兽药检测等方面已有应用^［18-23］^，并显示出了较好的净化效果。麦角生物碱毒素易溶于有机溶剂，适合于SLE的前处理净化，将SLE前处理技术结合UPLC-MS/MS用于牧草中麦角生物碱毒素的检测，能够实现高通量的快速准确测定。

本研究将SLE结合UPLC-MS/MS用于牧草中风险因子麦角生物碱毒素的快速检测，方法前处理操作简单，净化效果良好，环保节约，拓展了SLE方法的应用领域，可为监控牧草品质提供技术支持和保障。

## 1 实验部分

### 1.1 仪器与试剂

1260-6495超高效液相色谱-三重四极杆质谱仪（美国Agilent公司），ST16R冷冻离心机（美国Thermo公司），Vortex-Genie2涡旋振荡器（美国Scientific Industries），EFGC-11250氮吹仪（美国Orgamomatio公司），Qnintix313型电子天平（美国Sartorius公司）。

标准品双氢麦角汀、麦角柯宁碱、麦角异柯宁碱、麦角克碱、麦角异克碱、麦角卡里碱、麦角异卡里碱、麦角生碱、麦角醇、麦角胺购自阿尔塔公司（天津阿尔塔科技有限公司），纯度均大于98%。乙腈、甲醇、甲酸（formic acid，FA）、乙酸铵（色谱纯，上海安谱实验科技股份有限公司），二氯甲烷（分析纯，福晨（天津）化学试剂有限公司），乙酸乙酯、正己烷（HPLC级，武汉弗顿控股有限公司），甲基叔丁基醚（色谱级，上海阿拉丁生化科技股份有限公司）。实验用水为Milli-Q超纯水。SLE柱为Chem Elut柱（3 mL，美国Agilent公司）。0.22 μm聚四氟乙烯滤膜（上海安谱实验科技股份有限公司）。

苜蓿（alfalfa）购自美国Global Corniche International公司，燕麦（oats）和羊草（*Leymus chinensis*）为内蒙古自治区农牧业质量安全与检测研究所提供，青贮玉米（silage corn）取自鄂尔多斯市奶牛养殖场，其中苜蓿、燕麦和羊草均为干牧草。

### 1.2 标准溶液的配制

将各标准品用乙腈稀释成1.0 μg/mL的标准储备液，于冰箱4 ℃避光保存。使用时以乙腈-水（1∶3，体积比）混合成所需浓度的混合标准工作液，临用前逐级稀释成低浓度标准工作溶液。

### 1.3 样品前处理

将牧草样品用四分法缩至50~100 g，经粉碎机粉碎，通过1.0 mm孔径筛，混匀，将50 g试样置于500 mL广口瓶中密封，-18 ℃冷冻避光保存。准确称取2.0 g样品（精确至0.01 g）于50 mL聚乙烯具塞离心管中，加入8.0 mL乙腈（含1%甲酸），涡旋混匀1 min，振荡提取20 min，8 000 r/min 离心5 min，取上清液1.0 mL，加入3.0 mL水涡旋混匀0.5 min，混合液全部上SLE柱，静置10 min，使用6.0 mL乙酸乙酯洗脱，收集全部洗脱液，低于40 ℃下氮气吹干，用1 mL乙腈-水（1∶3，体积比，含0.5%甲酸）溶解残渣，涡旋0.5 min，经0.22 μm滤膜过滤后，上机测定。

### 1.4 色谱-质谱条件

Acquity UPLC BEH C_18_色谱柱（100 mm × 2.1 mm，1.7 μm）；流动相A：0.5%甲酸水溶液（含0.25 mmol/L乙酸铵），流动相B：0.5%甲酸乙腈溶液；梯度洗脱程序：0~1.0 min，90%B；1.0~5.0 min，90%B~75%B；5.0~10 min，75%B~50%B；10~12 min，50%B~10%B；12~14 min，10%B；14~16 min，10%B~90%B。流速：0.2 mL/min；进样量：10 μL。

电喷雾离子源，正离子扫描模式；检测方式为多反应监测（MRM）模式，干燥气温度为325 ℃；干燥气流量为11 L/min；雾化气压力为137.9 kPa；毛细管电压为4 000 V。其他参数见表1。

**表1:** 10种麦角生物碱毒素的质谱参数

Compound	Retention time/min	Precursor ion （*m/z*）	Product ions （*m/z*）	Collision energies/eV
Dihydroergocristine （双氢麦角汀）	8.94	612.5	350.0^*^， 271.5	30， 45
Ergocornine （麦角柯宁碱）	8.60	562.3	268.0^*^， 223.0	40， 45
Ergocorninine （麦角异柯宁碱）	9.11	562.3	305.0^*^， 223.0	30， 40
Ergocristine （麦角克碱）	8.94	610.0	268.0^*^， 223.0	30， 40
Ergocristinine （麦角异克碱）	9.42	610.0	268.0^*^， 223.0	30， 40
Ergocryptine （麦角卡里碱）	8.89	576.3	268.0^*^， 208.0	40， 50
Ergocryptinine （麦角异卡里碱）	9.31	576.3	268.0^*^， 223.0	30， 30
Ergosine （麦角生碱）	8.42	548.0	223.0^*^， 268.0	40， 30
Ergoitol （麦角醇）	6.42	254.8	240.0^*^， 44.0	20， 30
Ergotamine （麦角胺）	8.52	582.4	223.0^*^， 208.4	40， 50

* Quantitative ion.

## 2 结果与讨论

### 2.1 实验条件考察

#### 2.1.1 质谱条件的确定

将10种麦角生物碱毒素（1 μg/mL）的单标溶液在正、负离子模式下进行全扫描确定母离子。10种麦角生物碱毒素在正离子模式下响应值均高于负离子模式，因此选择扫描模式为正离子模式。对确定的母离子再进行子离子扫描确定子离子。对碰撞电压、干燥气温度、流速等进行优化，最终确定质谱参数（见表1）。

#### 2.1.2 色谱分离条件的优化

10种麦角生物碱毒素中含有3对同分异构体，分别是麦角柯宁碱和麦角异柯宁碱、麦角克碱和麦角异克碱、麦角卡里碱和麦角异卡里碱。为实现同分异构体的色谱分离，分别对色谱柱和流动相进行了优化。

首先对比了Acquity UPLC BEH C_18_（100 mm×2.1 mm，1.7 μm，美国Waters公司）、Acquity UPLC BEH Phenyl（100 mm×2.1 mm，1.7 μm，美国Waters公司）、Zorbax SB-C_18_（100 mm×2.1 mm，1.8 μm，美国Agilent公司）、Inertsil ODS-3（100 mm×2.1 mm，3.0 μm，日本GL Science公司）4种色谱柱。结果显示，Acquity UPLC BEH C_18_柱分离能力优于其他色谱柱，3对异构体能够基线分离；BEH Phenyl柱分离效果较差；Zorbax SB-C_18_柱和Inertsil ODS-3柱分离效果类似，可将麦角卡里碱和麦角异卡里碱分离，但对麦角柯宁碱和麦角异柯宁碱、麦角克碱和麦角异克碱分离效果较差（图1）。

**图1:**
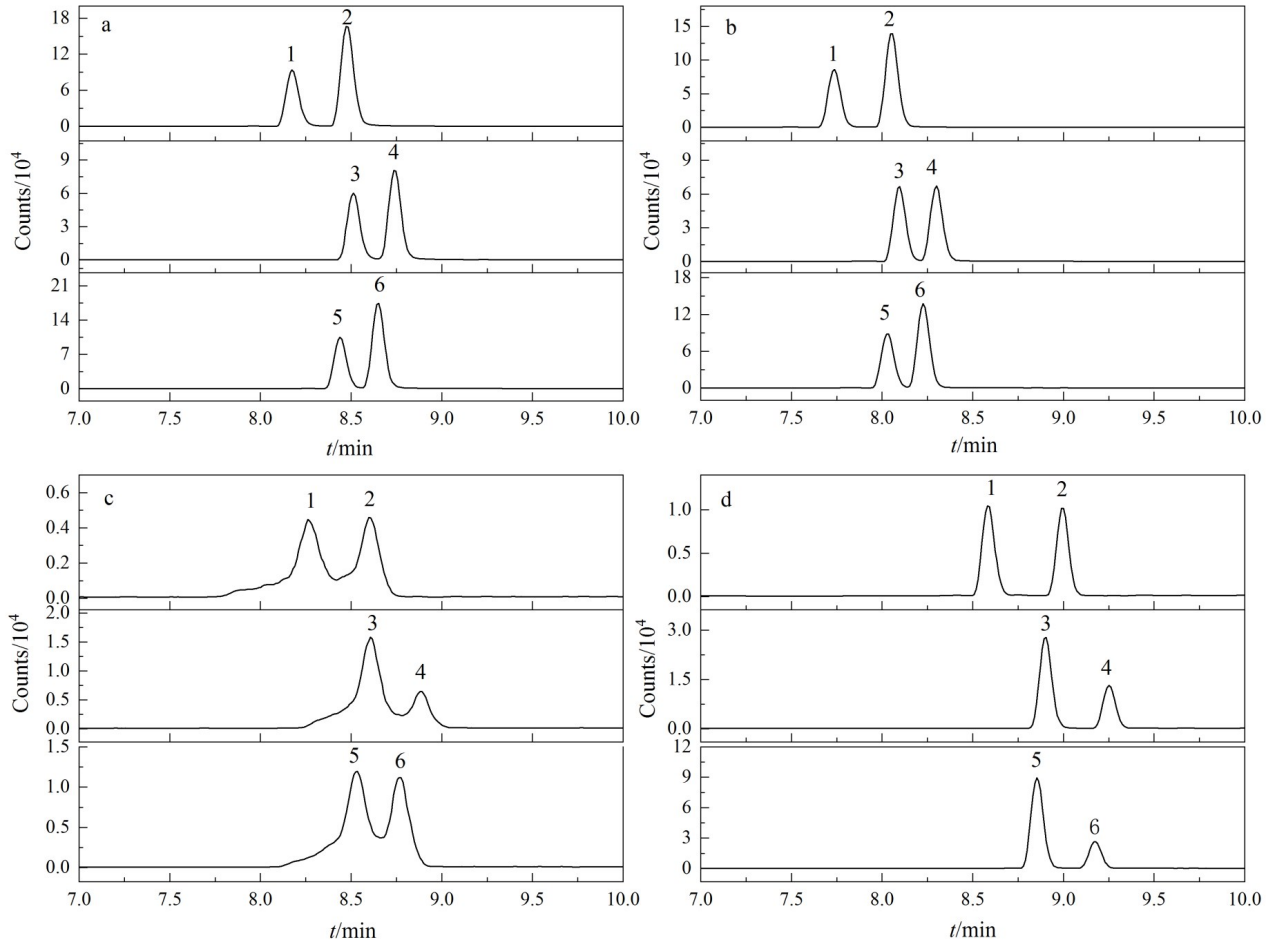
采用不同色谱柱时麦角生物碱毒素异构体的色谱图

对比了甲醇-水和乙腈-水两种流动相体系，结果表明用乙腈-水体系分离时，待测物分离度和峰形均优于甲醇-水体系。在电喷雾离子源的正离子扫描模式下，流动相中含有甲酸能够提高化合物的离子化效率，实验对比了水相和有机相中均含有0.1%~1.0%甲酸时待测物的响应变化。实验结果显示，0.5%的甲酸能够使待测物获得最优的响应，再提高甲酸体积分数待测物响应无明显提高。但在仅含甲酸的流动相体系中双氢麦角汀会有拖尾现象，进一步实验显示水相中加入乙酸铵能够抑制双氢麦角汀的拖尾，实验对比了水相中乙酸铵浓度（0.1~0.4 mmol/L）对待测物响应的影响。结果显示，0.25 mmol/L的乙酸铵能够保证化合物有较好的峰形且未明显降低响应值。因此最终确定流动相水相为0.5%甲酸水溶液（含0.25 mmol/L乙酸铵），流动相有机相为0.5%甲酸乙腈溶液。10种化合物的色谱分离情况见图2。

**图2:**
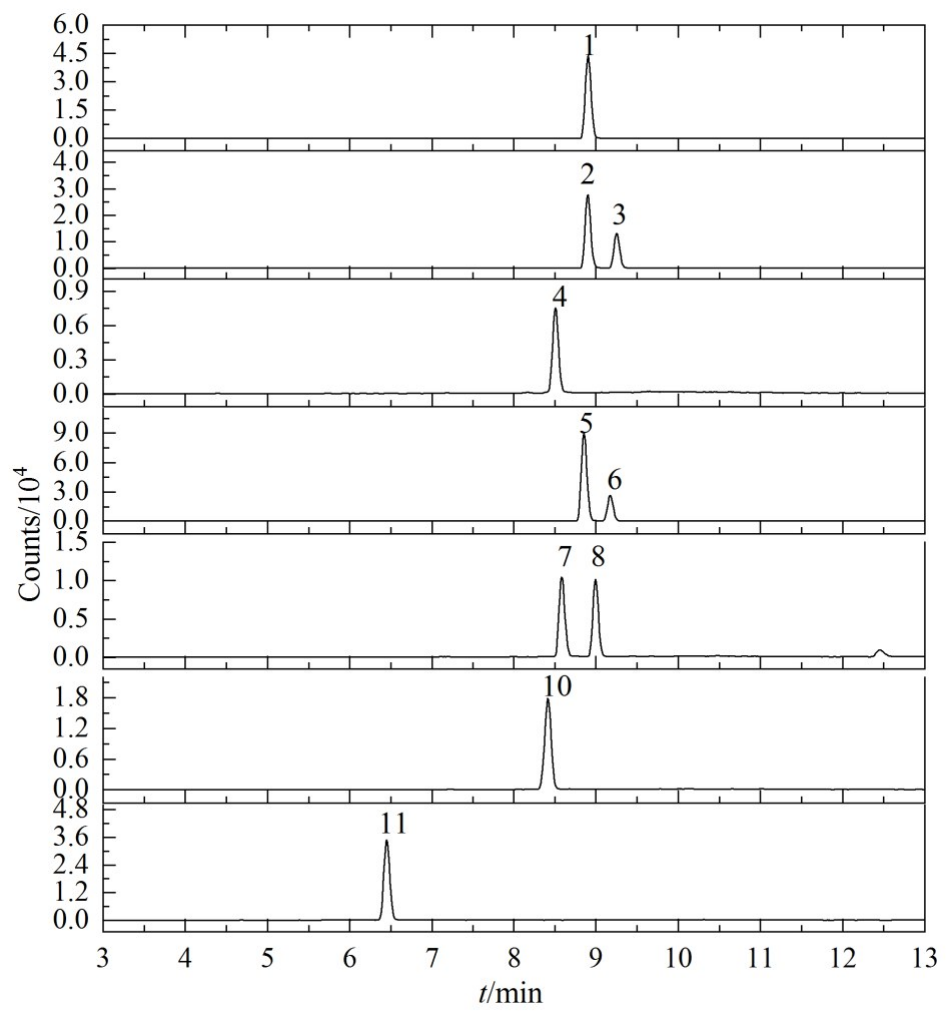
10种麦角生物碱毒素的色谱图

#### 2.1.3 提取溶剂的优化

霉菌毒素类的提取溶剂常采用甲醇、乙腈等，本实验研究了甲醇、乙腈、甲醇-水（7∶3，体积比）和乙腈-水（8∶2，体积比）的提取效率，以苜蓿为基质，添加麦角生物碱毒素标准物质，混匀后静置过夜，按照1.3节操作后测定。由图3a可见，整体提取效率：乙腈>乙腈-水（8∶2，体积比）>甲醇>甲醇-水（7∶3，体积比），这与乙腈极性较大、穿透能力强的特性有关，乙腈对10种组分的提取效率为61%~94%，因此综合考虑选用乙腈为提取溶剂。文献表明在提取溶剂中添加辅助试剂甲酸，能够增强提取的效果^［24］^，尝试在提取溶剂乙腈中加入0.5%、1%、2%甲酸进行提取，比较酸度对提取效率的影响（图3b）。大部分目标物的提取效率随酸的体积分数提高而提高，但体积分数高于1%后再无明显提升，并且其中麦角胺等化合物随甲酸含量的提高有所下降，因此选择1%甲酸乙腈为提取溶剂。

**图3:**
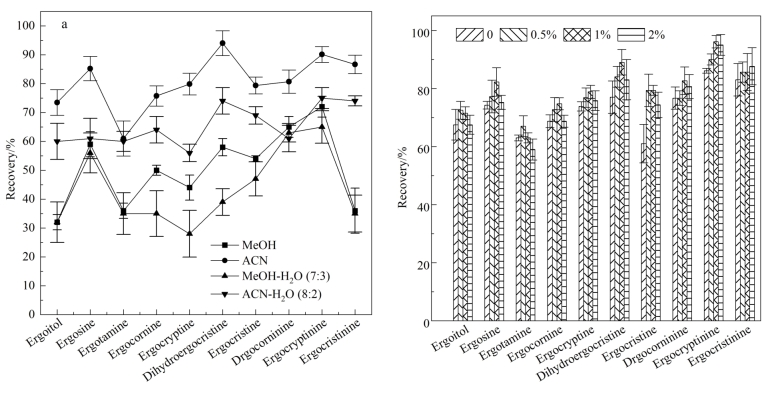
（a）提取溶剂类型和（b）乙腈中甲酸含量对10种麦角生物碱毒素回收率的影响（*n*=3）

#### 2.1.4 洗脱溶剂的选择

SLE步骤简洁，只需要上样和洗脱两步，洗脱溶剂是方法的关键部分。SLE与传统液液萃取技术在溶剂选择上遵循相同的原则：首先洗脱溶剂应不与上柱的提取液互溶，从而实现两相间的物质传递，其次洗脱溶剂有合适的极性能够对待测物有良好的溶解性^［17］^。比较了常用的二氯甲烷、乙酸乙酯、正己烷和甲基叔丁基醚4种洗脱溶剂（图4a）。结果显示，正己烷对10种麦角生物碱毒素的萃取效果均较差，萃取效率普遍低于20%，原因主要在于正己烷极性较低，对麦角生物碱毒素的溶解度低；二氯甲烷对麦角卡里碱、麦角异卡里碱、麦角克碱、麦角胺、麦角异柯宁碱的回收率均低于50%；甲基叔丁基醚和乙酸乙酯显示了相似的结果，优于正己烷和二氯甲烷。综上选择乙酸乙酯进行洗脱。

**图4:**
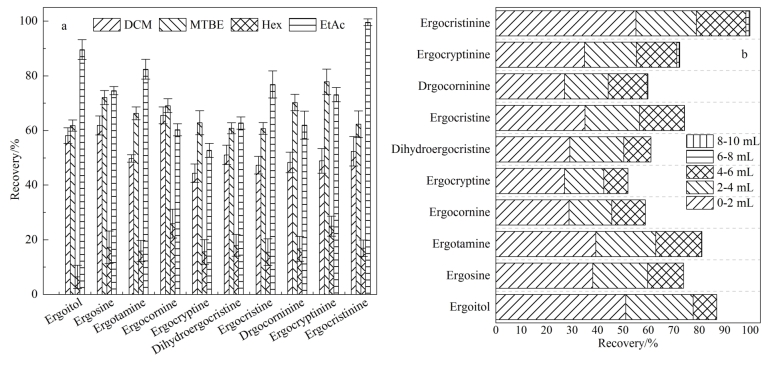
洗脱溶剂（a）类型和（b）体积对10种麦角生物碱毒素回收率的影响（*n*=3）

进一步通过分段接收的方式对洗脱溶剂的用量进行了考察，分段接收（每2 mL）洗脱液，测定回收率。结果显示麦角生物碱毒素的洗脱主要在前6 mL，超过6 mL后基本无明显洗脱物，考虑到进一步增加洗脱溶剂用量会导致共洗脱的杂质增多，基质响应增强，因此选择洗脱溶剂用量为6 mL（图4b）。

### 2.2 基质效应

基质效应（ME）主要是化合物在质谱离子源离子化时产生，样品基质中的共流出杂质组分在离子化过程中对目标物的响应值有增强或抑制的作用，这种干扰影响就是基质效应^［25，26］^，基质效应会影响定量分析结果的准确性。ME定义为［（基质标准曲线线性方程斜率/溶剂标准曲线线性方程斜率）-1］×100%。∣ME∣≥50%为强基质效应，20%<∣ME∣<50%为中等基质效应，∣ME∣≤20%为弱基质效应。

麦角生物碱毒素在牧草中的基质效应研究尚未见报道，本研究选取常见的苜蓿、燕麦、羊草和青贮玉米4种牧草种类作为基质，研究了麦角生物碱毒素在其中的基质效应。结果表明牧草中的基质效应多表现为基质抑制，其中青贮玉米的基质效应显著强于其他3种牧草，这可能是由于发酵后基质更为复杂的原因，其中50%以上为强基质效应。苜蓿、燕麦和羊草无强基质效应，相对基质效应较弱，以弱基质效应为主，其中弱基质效应占比高于63%（表2）。鉴于仍有部分基质无法避免基质效应，因此采用基质标准曲线外标法定量以降低基质效应。

**表 2:** 牧草中麦角生物碱毒素的基质效应

Mycotoxin	Matrix effects/%			
	Alfalfa	*Leymus chinensis*	Oats	Silage corn
Ergoitol	-8.7	-8.5	-0.6	-1.2
Ergosine	-10.2	-28.1	-19.2	-41.2
Ergotamine	-9.4	-26.2	-22.6	-57.6
Ergocornine	-1.3	-16.7	-13.7	-48.7
Ergocryptine	-20.3	-33.5	-33.2	-59.8
Dihydroergocristine	-9.4	-24.8	-26.0	-64.7
Ergocristine	6.6	-9.9	-6.6	-48.9
Ergocorninine	3.3	-9.1	-9.9	-20.6
Ergocryptinine	28.4	-0.7	-19.7	-63.0
Ergocristinine	49.1	32.7	11.2	-55.4

### 2.3 方法学考察

#### 2.3.1 标准曲线及检出限

将不含待测物的牧草样品按照优化的方法处理得到空白基质提取液，用以配制基质标准工作溶液。以各麦角生物碱毒素的质量浓度为横坐标（*x*），定量离子峰面积为纵坐标（*y*），绘制标准曲线，结果表明，10种麦角生物碱毒素标准曲线均具有良好的线性关系，线性相关系数（*r* ^2^）＞0.995（表3）。根据各目标化合物在谱图上的出峰高度和出峰附近的平均噪声高度计算各化合物的信噪比，以3倍信噪比和10倍信噪比时目标化合物定量离子峰面积对应的质量浓度为检出限（LOD）和定量限（LOQ）。10种麦角生物碱毒素的检出限为0.1~2.3 μg/kg，定量限为0.4~7.3 μg/kg。

**表 3:** 10种麦角生物碱毒素的线性范围、线性方程、相关系数、检出限及定量限

Analyte	Linear range/（ng/mL）	Matrix	Linear equation	*r* ^2^	LOD/（µg/kg）	LOQ/（µg/kg）
Ergoitol	0.02-10	A	*y*=406187.6*x*-5321.3	0.9983	0.2	0.5
		L	*y* =407123.8*x*-3593.5	0.9995	0.3	0.8
		O	*y* =441943.1*x*-5575.1	0.9963	0.1	0.5
		S	*y* =439285.3*x*-3960.3	0.9995	0.2	0.6
Ergosine	0.02-10	A	*y* =223583.7*x*-2592.8	0.9977	0.1	0.5
		L	*y* =179002.7*x*+283.8	0.9963	0.1	0.4
		O	*y*=201027.6*x*-1034.5	0.9984	0.1	0.5
		S	*y*=146432.1*x*-1395.9	0.9978	0.3	0.5
Ergotamine	0.02-10	A	*y*=17602.2*x*-1740.4	0.9956	0.2	1.0
		L	*y*=14351.0*x*-107.5	0.9974	0.8	2.2
		O	*y*=15047.4*x*-227.4	0.9983	0.8	2.0
		S	*y*=8232.8*x*+186.7	0.9998	0.6	1.7
Ergocornine	0.05-20	A	*y*=241847.0*x*-3757.3	0.9967	0.4	1.0
		L	*y*=204177.0*x*-2390.6	0.9990	0.9	3.0
		O	*y*=211635.7*x*-858.7	0.9996	0.9	2.3
		S	*y*=125799.3*x*-1575.6	0.9994	1.0	2.4
Ergocryptine	0.2-50	A	*y*=402484.5*x*-6950.3	0.9956	0.8	1.9
		L	*y*=335757.6*x*-1754.8	0.9992	1.3	4.1
		O	*y*=337455.4*x*-68.7	0.9999	1.0	3.2
		S	*y*=202891.5*x*-2736.9	0.9976	1.8	4.0
Dihydroergocristine	0.2-50	A	*y*=98988.2*x*-10794.2	0.9953	1.6	3.3
		L	*y*=82217.9*x*-6029.7	0.9954	1.9	4.5
		O	*y*=80918.5*x*-4153.9	0.9965	2.0	4.1
		S	*y*=38582.2*x*+3290.0	0.9961	2.2	6.2
Ergocristine	0.05-20	A	*y*=138974.5*x*-10782.5	0.9968	0.4	1.8
		L	*y*=117374.6*x*-2056.8	0.9984	0.9	4.2
		O	*y*=121682.2*x*-4690.4	0.9996	0.6	2.8
		S	*y*=66665.5*x*-3204.4	0.9991	2.3	7.3
Ergocorninine	0.05-20	A	*y*=245674.0*x*-3245.4	0.9976	0.3	0.9
		L	*y*=216163.8*x*-2039.6	0.9990	0.4	0.7
		O	*y*=214161.6*x*-2267.5	0.9972	0.3	0.8
		S	*y*=188711.3*x*-245.5	0.9978	0.4	0.9
Ergocryptinine	0.05-20	A	*y*=20416.4*x*-2344.6	0.9978	0.5	2.1
		L	*y*=15801.0*x*-900.0	0.9991	0.6	2.3
		O	*y*=12773.4*x*-544.7	0.9963	0.7	2.7
		S	*y*=5886.7*x*-338.9	0.9998	1.0	4.5
Ergocristinine	0.2-50	A	*y*=494653.8*x*-9909.0	0.9953	1.3	5.5
		L	*y*=440326.7*x*-6537.3	0.9974	1.6	4.6
		O	*y*=368928.4*x*-3445.5	0.9959	1.3	3.3
		S	*y*=147932.6*x*-1244.7	0.9995	1.4	4.9

A： alfalfa； L： *Leymus chinensis；* O： oats； S： silage corn. *y*： peak areas of analyte； *x*： mass concentration， ng/mL.

#### 2.3.2 回收率与精密度

依照建立的方法对4种空白样品（苜蓿、羊草、燕麦、青贮玉米）进行三水平加标试验，每个添加水平取6个平行样，添加水平分别为1、2、10倍LOQ。方法的回收率与精密度数据见表4。方法的回收率为66.3%~116.7%，相对标准偏差为1.1%~9.9%，重复性和稳定性较好，满足残留分析的要求。

**表 4:** 牧草样品中10种麦角生物碱毒素的回收率及相对标准偏差（*n=*6）

Analyte	Matrix	1-fold LOQ		2-fold LOQ		10-fold LOQ	
		Recovery/%	RSD/%	Recovery/%	RSD/%	Recovery/%	RSD/%
Ergoitol	A	76.9	5.4	80.3	3.8	84.3	4.1
	L	88.5	2.7	91.2	3.3	92.3	4.3
	O	77.7	6.6	80.6	5.1	82.8	5.0
	S	72.0	7.6	74.0	6.2	81.9	6.5
Ergosine	A	68.3	8.9	77.6	3.2	81.1	3.1
	L	68.9	7.3	72.4	3.6	80.6	3.8
	O	69.4	7.0	72.0	5.7	76.4	1.2
	S	74.4	4.4	75.0	4.1	76.7	3.7
Ergotamine	A	74.2	8.2	76.7	5.3	85.4	6.4
	L	72.1	8.1	75.4	6.5	86.4	3.4
	O	70.2	7.7	74.5	5.7	77.9	5.1
	S	71.6	7.2	79.3	6.2	75.9	5.9
Ergocornine	A	82.1	9.9	84.3	8.1	85.6	2.8
	L	79.6	9.2	82.4	6.4	88.4	1.4
	O	78.6	5.6	81.9	3.7	84.2	5.5
	S	85.5	4.8	93.1	4.8	99.9	4.1
Ergocryptine	A	73.6	9.1	75.8	9.7	78.9	6.7
	L	70.6	6.8	77.4	3.3	80.6	4.6
	O	72.4	7.1	79.0	5.3	77.7	3.7
	S	70.1	7.6	74.2	7.0	80.0	6.8
Dihydroergocristine	A	66.9	9.6	75.8	3.6	77.5	3.2
	L	66.4	3.3	74.1	3.6	79.8	3.9
	O	74.5	5.3	77.8	6.5	81.0	3.0
	S	70.3	7.3	70.5	7.4	76.1	7.1
Ergocristine	A	78.4	7.9	79.2	5.4	82.3	2.4
	L	70.4	9.2	75.3	6.5	80.2	1.1
	O	70.3	5.8	71.4	4.5	74.8	3.2
	S	74.7	6.1	79.2	7.1	82.1	5.9
Ergocorninine	A	73.7	3.2	73.0	2.8	75.6	1.7
	L	86.1	2.6	90.2	4.5	108.3	1.3
	O	108.6	7.6	111.0	4.9	116.2	2.2
	S	95.9	4.8	97.2	2.8	116.7	3.8
Ergocryptinine	A	66.3	5.6	72.4	5.7	73.5	5.0
	L	73.3	9.2	76.7	6.1	96.5	5.6
	O	76.7	8.4	78.0	5.2	81.9	1.1
	S	72.3	7.0	79.0	5.8	87.7	5.8
Ergocristinine	A	87.1	5.4	89.3	7.3	92.3	5.2
	L	74.5	1.9	78.7	2.4	94.7	4.2
	O	74.4	4.3	80.5	4.7	79.1	3.2
	S	79.3	6.0	86.1	6.1	94.6	5.6

### 2.4 实际样品检测

采用所建立的方法对4批进口苜蓿和两批鄂尔多斯市奶牛饲养场的青贮牧草和混合牧草进行检测，在1份混合牧草中检出麦角克碱11.3 μg/kg（RSD=2.9%，*n*=3），其他麦角生物碱毒素均未检出，阳性样品按10.0 μg/kg 加标水平进行加标回收试验，回收率为87.6%（RSD=1.3%，*n*=3），符合质控要求，方法可用于实际样品分析。阳性样品色谱图见图5。

**图 5:**
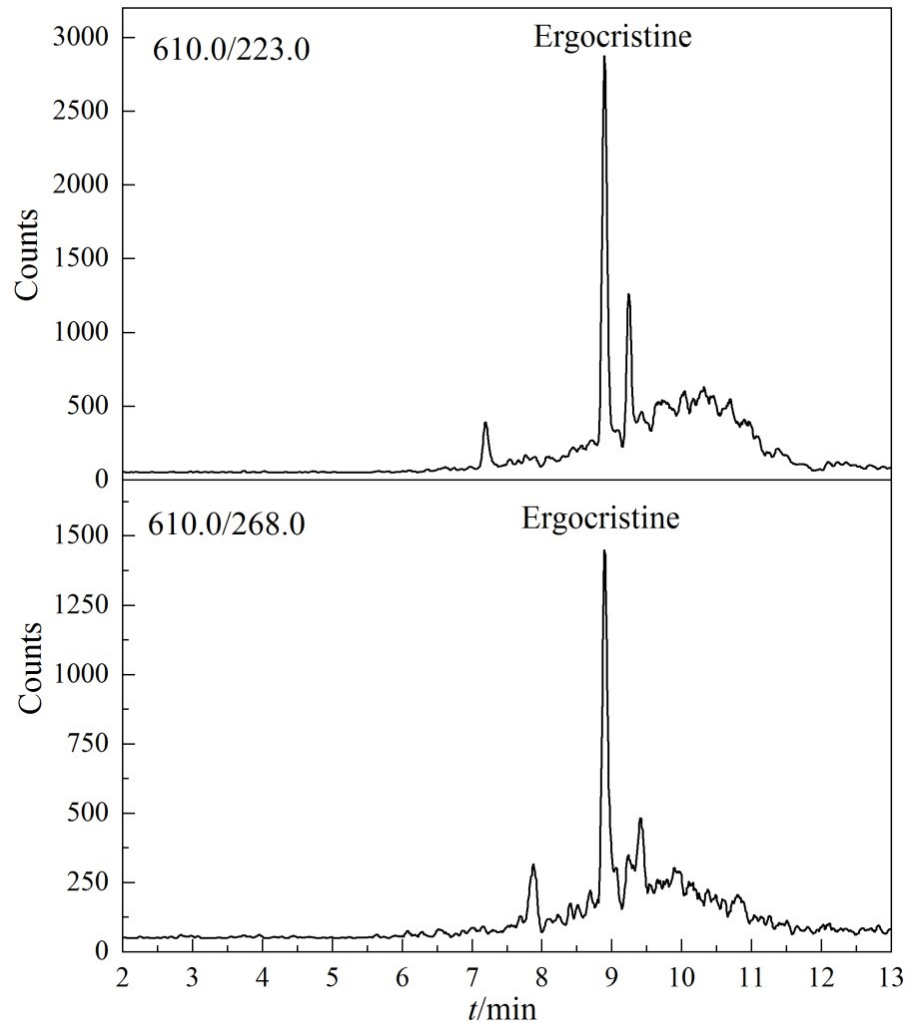
阳性样品的MRM色谱图

## 3 结论

本研究针对牧草样品，采用固相支撑液液萃取技术对样品进行处理，结合超高效液相色谱-串联质谱，建立了10种麦角生物碱毒素的快速检测方法。本方法操作简单，结果稳定，基质效应较低，重复性好，适用于多种牧草中麦角生物碱毒素的准确定量。方法兼具简洁高效和环保的特点，可为监控牧草品质提供技术支持，同时拓展了固相支撑液液萃取技术在毒素检测领域的应用。
